# Insulin-like growth factor-binding protein-3 is induced by tamoxifen and fulvestrant and modulates fulvestrant response in breast cancer cells

**DOI:** 10.3389/fonc.2024.1452981

**Published:** 2024-11-15

**Authors:** Keenan L. Flynn, Yan Zheng, Janel Y. Sowers, Nefretiri J. T. Masangya, Kevin D. Houston

**Affiliations:** Department of Chemistry and Biochemistry, New Mexico State University, Las Cruces, NM, United States

**Keywords:** GPER1, Yap/Taz, fulvestrant, triple negative breast cancer, IGFBP-3, insulin like growth factor binding protein

## Abstract

**Introduction:**

Insulin-like growth factor binding protein-3 (IGFBP-3) exerts varying effects on estrogen receptor alpha (ERα)-positive and triple-negative breast cancer (TNBC) cells. In ERα-positive cells, IGFBP-3 is antiproliferative and proapoptotic. In contrast, IGFBP-3 stimulates proliferation in triple-negative breast cancer (TNBC) cells via EGFR activation.

**Methods:**

To identify potential mechanisms that underlie the opposing effects of IGFBP-3 on these two breast cancer subtypes, IGFBP-3 expression was determined in cell line models of both ERα-positive breast cancer and TNBC, and cells were treated with antiestrogens tamoxifen and fulvestrant.

**Results and discussion:**

MCF-7 and T-47D cells expressed low levels of IGFBP-3 when compared to MDA-MB-231 and MDA-MB-468 cells. MCF-7 cells with acquired resistance to the selective estrogen receptor degrader fulvestrant expressed high IGFBP-3 and MCF-7 cells with constitutive IGFBP-3 expression were fulvestrant resistant. IGFBP-3 expression was increased in all cell lines upon treatment with fulvestrant or the selective estrogen receptor modulator tamoxifen and both fulvestrant and tamoxifen increased TNBC cell proliferation. Further, IGFBP-3 expression was increased by treatment with the GPER1 agonist G-1 and attenuated upon treatment with P17, a YAP/TAZ inhibitor. These data suggest that IGFBP-3 modulates breast cancer cells and is a mediator of breast cancer cell response to fulvestrant and tamoxifen.

## Graphical Abstract

Graphical abstract of proposed mechanism of IGFBP-3 induction by GPER1 agonists. Antiestrogens tamoxifen (Tam) and fulvestrant (Ful) inhibit the actions of estrogen receptor alpha (ERα) but activate the g-protein coupled estrogen receptor (GPER1), similar to G-1, the selective GPER1 agonist. GPER1 activates transcriptional coactivators YAP and TAZ, which induce the expression of IGFBP-3. Once induced, IGFBP-3 promotes survival and proliferation in triple-negative breast cancer cells. While Tam and Ful both induced IGFBP-3, YAP/TAZ were not involved in Ful-induced IGFBP-3 expression, suggesting alternative signaling pathways that induce IGFBP-3 expression in response to Ful.

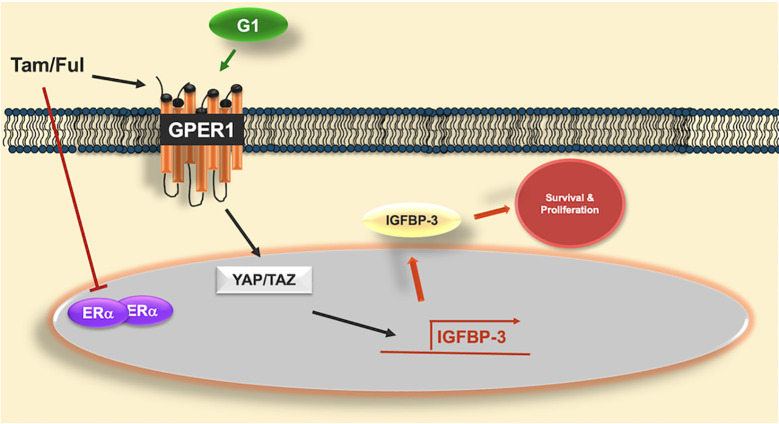

## 1 Introduction

The insulin-like growth factor (IGF) system plays essential roles in many cellular processes and is an important growth factor in breast cancer (1). The IGF system is activated upon binding of ligands (i.e. IGF-1) to transmembrane receptors (i.e. IGF-1R) and is modulated by six IGF binding proteins (IGFBP-1 to 6) (2). Of the six IGFBPs, IGFBP-3 is the most well-characterized and has been shown to bind over 90% of circulating IGF-1 in serum (3). IGFBP-3-bound IGF-1 is both stabilized resulting in increased half-life and sequestered to prevent binding to IGF-1R, thus inhibiting activation of the IGF system (4, 5). In addition to its IGF-dependent functions, IGFBP-3 is reported to directly bind the low-density lipoprotein receptor-related protein 1 (LRP1), type V TGFβ receptor, and a novel cell death receptor later named IGFBP-3R, all of which are suggested to play an antiproliferative role (6). IGFBP-3 is a secreted protein with extracellular functions but also translocates to the nucleus via its nuclear localization sequence (NLS) domain. When nuclear, IGFBP-3 binds to retinoid X receptor a (RXRa), resulting in corepressor dissociation and transcriptional activation of target genes (7).

In ERα positive breast cancer cells, IGFBP-3 is antiproliferative after exogenous expression or treatment (8, 9). Additionally, Li et al. demonstrated that IGFBP-3 attenuates antiestrogen resistance in fulvestrant-resistant MCF-7 cells by interacting with GPR78-caspase 7 complex, thus resulting in activation of caspase 7 and apoptosis (10). Other reports indicate that IGFBP-3 binds to GPR78 to stimulate autophagy, thus promoting survival in ERα positive breast cancer cells (11, 12). A proliferative role for IGFBP-3 is observed in triple-negative breast cancer (TNBC) cells via modulating sphingosine kinase-1 (SphK1) localization to activate EGFR signaling (13, 14). Clinically, higher IGFBP-3 is observed in malignant breast tumors compared to their benign counterparts (15). Further, higher IGFBP-3 in breast tumors is associated with worsened relapse-free breast cancer patient survival (16). These findings and the documented actions of IGFBP-3 on SphK1/EGFR signaling illustrate the tumor-promoting character of the protein, but conflict with other literature that documents a tumor-suppressive role for the ERα positive subtype. While IGFBP-3 is clearly an important modulator of breast cancer, mechanisms of breast cancer cell modulation by IGFBP-3 are not adequately understood.

To identify mechanisms that underlie the opposing effects that IGFBP-3 has in breast cancer, the expression of IGFBP-3 was measured in cell line models of ERα positive and triple-negative breast cancer (TNBC) cells. MCF-7 and T-47D cells expressed low but detectable IGFBP-3 (intracellular and extracellular), whereas both MDA-MB-231 and MDA-MB-468 cells had significantly higher levels of IGFBP-3 when compared to ERα positive breast cancer cells. Stable expression of IGFBP-3 in MCF-7 breast cancer elicited resistance to the selective estrogen receptor degrader (SERD) fulvestrant (Ful), and MCF-7 cells with acquired resistance to Ful (MCF-7^FulR^) have higher IGFBP-3 expression than parental cells. Upon treatment with Ful, IGFBP-3 was induced in breast cancer cells similar to data observed in reports describing the regulation of IGFBP-1 by the G-protein-coupled estrogen receptor (GPER1) and clinical studies showing IGFBP-3 serum increases in response to Ful (17–19). Tamoxifen (Tam), a selective estrogen receptor modulator (SERM), also increased IGFBP-3 expression. As both antiestrogens have agonistic effects on GPER1, TNBC cells were treated with G-1, a specific GPER-1 agonist. G-1 treatment induced IGFBP-3 expression through activation of YAP/TAZ, suggesting that the observed IGFBP-3 accumulation is mediated by GPER-1. Consistent with this mechanism, inhibition of YAP/TAZ reduced Tam-induced IGFBP-3 expression. However, the same did not apply to Ful-induced expression, suggesting that Tam and Ful may induce IGFBP-3 expression by alternate mechanisms. Taken together, these data identify a novel mechanism of IGFBP-3 regulation in breast cancer cells and provide evidence for an association between IGFBP-3 and response to antiestrogen therapies.

## 2 Methods

### 2.1 Cell culture and treatment

MCF-7 and T-47D ERα positive breast cancer cells and MDA-MB-231 and MDA-MB-468 TNBC cells were purchased from ATCC (ATCC, Manassas, VA). All cell lines were cultured in DMEM supplemented with 10% fetal bovine serum, 1 mM sodium pyruvate and 2 mM L-glutamine (Life Technologies, Carlsbad, CA). All cell lines were maintained in maintenance media and cells used in experiments were below passage 35. For Ful treatment, cells at 80% confluency were washed with 1X PBS and cultured in DMEM containing 10% charcoal stripped FBS for 24 hours. Cells were then washed with 1X PBS and treated with vehicle, DMSO, in serum-free media. For G-1 and G-1/P-17 treatment, the cells were washed with 1X PBS and serum starved using DMEM with 10% Charcoal Stripped FBS for 24 hours. After the 24 hours, the cells were washed with 1X PBS and treated with vehicle (DMSO) G-1 and/or P-17 in serum-free DMEM for 24 hours.

### 2.2 Establishment of Ful-resistant MCF-7 cells (MCF-7 ^FulR^)

Ful resistant MCF-7 cells were established using a previously described method used for Tam (20). MCF-7 cells were exposed to 1 µM Ful in maintenance media and fresh media containing Ful was added every 3 days. After 21 days of Ful exposure, cells that remained were allowed to recover and grow in fresh maintenance media. Cells were then split and maintained in media containing 1 µM Ful and designated MCF-7 FulR.

### 2.3 Establishment of stable cell lines MCF-7-EV and MCF-7-BP3

Stable subclones of MCF-7 cells were generated following manufacturer protocols from the Lipofectamine 3000 transfection kit (Life Technologies, Carlsbad, CA). Human IGFBP-3 expression vector (NM_001013398) and the empty vector lacking the IGFBP-3 ORF were purchased from OriGene (Rockville, MD). Following a 96-hour transfection period in Opti-MEM (Life Technologies, Carlsbad, CA), cells were washed with 1X PBS and allowed to recover in maintenance media. Selection of stably transfected cells was accomplished by treatment with 800 μg/mL Geneticin (Life Technologies, Carlsbad, CA). Cells treated with neither EV nor IGFBP-3 expression vectors were also subjected to Geneticin treatment until all cells had perished. Cells were washed with 1X PBS and given fresh media with Geneticin every five days while non-treated cells perished. The remaining cells were transferred to T-75 flasks and allowed to grow in media containing 400 μg/mL G418. Validation of over-expression of IGFBP-3 in MCF-7-BP3 was accomplished with both Western Blotting and RT-qPCR.

### 2.4 Total RNA extraction and quantitative real-time PCR analysis

Total RNA was extracted and isolated with the PureLink RNA Mini Kit (Life Technologies, Carlsbad CA) followed by on-column DNA digestion using PureLink DNase Set (Life Technologies, Carlsbad CA). cDNA was synthesized from 1μg total RNA using the High-Capacity RNA-to-cDNA Kit (Life Technologies, Carlsbad CA) and used as template in subsequent quantitative real-time PCR (RT-qPCR) reactions. qRT-PCR was performed using SYBR Green Master Mix (Life Technologies, Carlsbad CA) and the 7300 Real-Time PCR system (Bio-Rad, Hercules, CA). Primer pairs used for qRT-PCR: human IGFBP-3 forward 5′-AGC-ACA-GAT-ACC-CAG-AAC-TTC-TCC-3′; reverse 5′-TCC-ATT-TCT-CTA-CGG-CAG-GG-3′. Human RPL30 gene was used as the internal control to normalize for mRNA in qRT-PCR reactions. Human RPL30 forward 5′-ACA-GCA-TGC-GGA-AAA-TAC-TAC-3′; reverse 5′-AAA-GGA-AAA-TTT-TGC-AGG-TTT-3′.

### 2.5 Immunoblot analysis

Cells were harvested with RIPA lysis buffer (Prod# 89901) containing protease and phosphatase inhibitor cocktails (Prod# 1862209 and 186249, Thermo Scientific, Rockford, IL). After lysis, cells were centrifuged at 12,000 x g for 15 minutes at 4°C, supernatant was collected, and protein concentrations was determined using BCA assay (Thermo Scientific, Rockford, IL). Equivalent masses of 30-75 μg total protein per lane were resolved using Bolt 4–12% Bis-Tris Plus gels and transferred to PVDF membrane (Life Technologies, Carlsbad, CA). PVDF membranes were blocked in 1X Tris-buffered saline-0.1% Tween 20 (TBST) containing 5% fat-free milk at room temperature for 1 hour with slow agitation. Membranes were washed with 1X TBST three times and primary antibody was added and incubated overnight at 4°C. The following primary antibodies including dilution factor in 5% milk TBST were used in the current study: IGFBP-3 (#25864, Cell Signaling Technology, Danvers, MA); β-actin (sc-47778, Santa Cruz Biotechnology, Dallas, TX). The dilution ratio for primary antibodies from Cell Signaling Technology was 1:1000; The dilution ratio for primary antibodies from Santa Cruz Biotechnology was 1:2000. After primary antibody incubation, membranes were washed three times with 1X TBST then incubated with anti-rabbit IgG conjugated to horseradish peroxidase (# 7074S, Cell Signaling Technology, Danvers, MA) or anti-mouse IgG conjugated to horseradish peroxidase (sc-81178, Santa Cruz Biotechnology, Dallas, TX) with dilution ratio of 1:5000 at room temperature for 1 hour. After washing membranes with 1X TBST three times, chemiluminescence reagent (34076, Thermo Scientific, Rockford, IL) was added and detected using Gel Doc™ XR ChemiDoc™ imaging system (BioRad, Hercules, CA) followed by quantification using ImageJ (NIH). Restore plus western blot buffer (46430, Thermo Scientific, Rockford, IL) was used to strip membranes of antibodies prior to probing for loading control.

### 2.6 Extracellular IGFBP-3 measurement

Conditioned culture media was concentrated as previously described (17). Briefly, media was collected and concentrated with centrifugal filter units (UFC800396, MillporeSigma, Burlington, MA) at 4°C with the speed of 4000 rpm for 1 hour. Concentrated media was collected with an addition of protease inhibitor cocktail (Prod #1862209, Thermo Scientific, Rockford, IL) and protein concentration was determined using BCA assay. Evidence for equivalent protein loading was obtained by Coomassie blue stain of gel and imaged using FOTODYNE gel imager (FOTODYN INCORPORATED, Hartland, WI).

### 2.7 IGFBP-3 ELISA

Extracellular IGFBP-3 was measured from conditioned media samples using Human IGFBP-3 ELISA Kit (Thermo Scientific, Carlsbad, CA, Cat No. EHIGFBP3) following the included protocol. Media was diluted by a factor of 1 for MCF-7 and T-47D samples, and by a factor of 5 for MDA-MB-231 and MDA-MB-468 samples in anticipation of high [IGFBP-3]. Absorbance values were recorded at 450 nm and IGFBP-3 concentrations were determined from the standard curve.

### 2.8 Cell viability assay

Cells were cultured in 10% charcoal-stripped DMEM for 24 hours then switched to serum-free DMEM with the indicated treatment. After 5 days of treatment, cells were trypsinized and harvested with 1X PBS and counted using a hemocytometer.

### 2.9 Expression and survival analysis of breast cancer patient tumor data

Assessment of the effects of IGFBP-3 expression on breast cancer patient survival was conducted using the KMplot.com free online resource (21). Trichotomization of the samples (Q1 vs Q4) was used to stratify the patient data by high versus low IGFBP-3 expression. For comparison of IGFBP-3 expression across multiple subtypes of breast cancer, the cBioportal METABRIC clinical dataset was used (22). ER+ breast cancer was defined as the ER+/HER2- high prolif and the ER+/HER2- low prolif groups on the 3-gene classifier. TNBC was defined as the ER-/HER2- group.

### 2.10 Statistical analysis

All statistical analysis was performed by one-way ANOVA with *post-hoc* Tukey’s tests using Prism 6 (GraphPad, San Diego, CA). Differences were considered significant if p ≤ 0.05 and the error bars are ± SEM.

## 3 Results

### 3.1 IGFBP-3 expression is higher in TNBC cells relative to ERα-positive breast cancer cells

**Figure 1:**
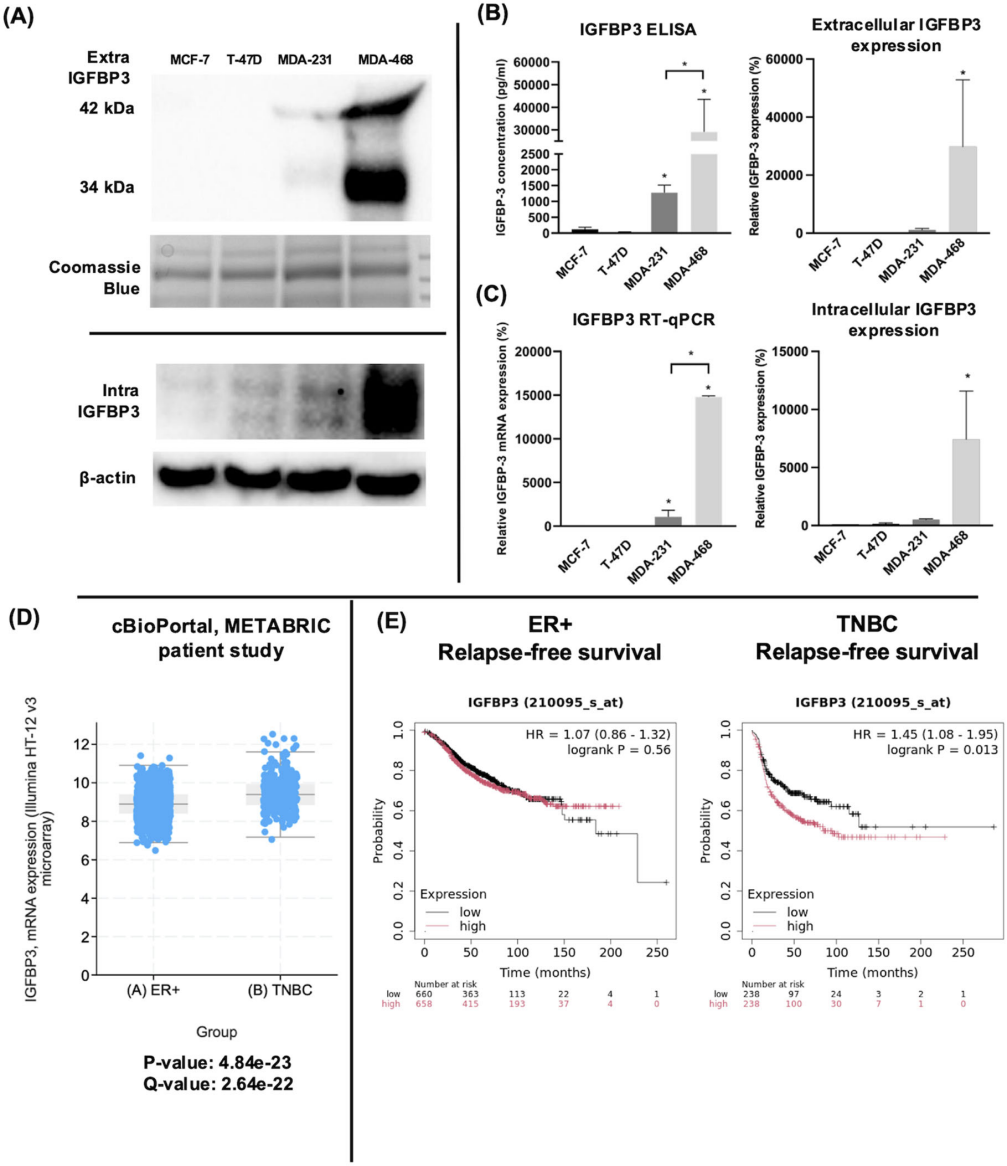
TNBC MDA-MB-231 and MDA-MB-468 cells expressed higher IGFBP-3 than ERα positive MCF-7 and T-47D cells. **(A)** immunoblot of extracellular and intracellular IGFBP-3 expression in MCF-7, T-47D, MDA-MB-231, and MDA-MB-468 cells; **(B)** ELISA analysis of conditioned media collected from the indicated cell lines to determine IGFBP-3 protein concentration; **(C)** quantitative real-time PCR analysis of IGFBP-3 mRNA levels. The IGFBP-3 mRNA level of MCF-7 is set as 100%. Results are representative of 3 independent experiments. **(D)** Comparison of IGFBP-3 mRNA expression between clinical patient cohorts in the METABRIC patient data set on cBioPortal; **(E)** Kaplan-Meier plots comparing low to high IGFBP-3 expression in ER+ and TNBC patient cohorts using the KMplot.com online resource. For 1D, p and q-values are reported; for 1E, logrank P values generated by the KMplot resource are provided. Extra-IGFBP-3, extracellular IGFBP-3; intra-IGFBP-3, intracellular IGFBP-3. Coomassie blue staining indicates the loading of concentrated media from different cell lines. Bar graph result is the average of 3 independent experiments, and error bars are the standard error of the mean. *p < 0.05.

The endogenous expression of IGFBP-3 was profiled in four breast cancer cell lines representing ERα positive and triple-negative clinical subtypes. Immunoblot and ELISA analysis of concentrated conditioned media from these cell lines indicated that the TNBC cell line MDA-MB-468 had the highest level of intra- and extracellular IGFBP-3 (**Figures 1A, B**). Because IGFBP-3 exists in multiple glycosylated forms, bands at 34 kDa and 42 kDa are observed in the immunoblot, while a doublet is observed inside the cell at 42 kDa (23). MCF-7 and T-47D cells expressed relatively low levels of IGFBP-3 and MDA-MB-231 cells had higher levels (**Figure 1A**). Consistent with the observations from immunoblot and ELISA analysis, MDA-MB-468 cells also had the highest level of IGFBP-3 mRNA, MDA-MB-231 cells had significantly less, and in MCF-7 and T-47D cells, IGFBP-3 mRNA was essentially undetectable by qPCR (**Figure 1C**). Corroborating these observations, elevated IGFBP-3 mRNA is observed in TNBC relative to ER+ breast cancer cells (**Figure 1D**). Additionally, high IGFBP-3 expression significantly worsens patient relapse-free survival compared to low expression (**Figure 1E**) and this was not observed in patients with ER+ breast tumors.

### 3.2 Elevated expression of IGFBP-3 is associated with fulvestrant resistance in MCF-7 cells

**Figure 2:**
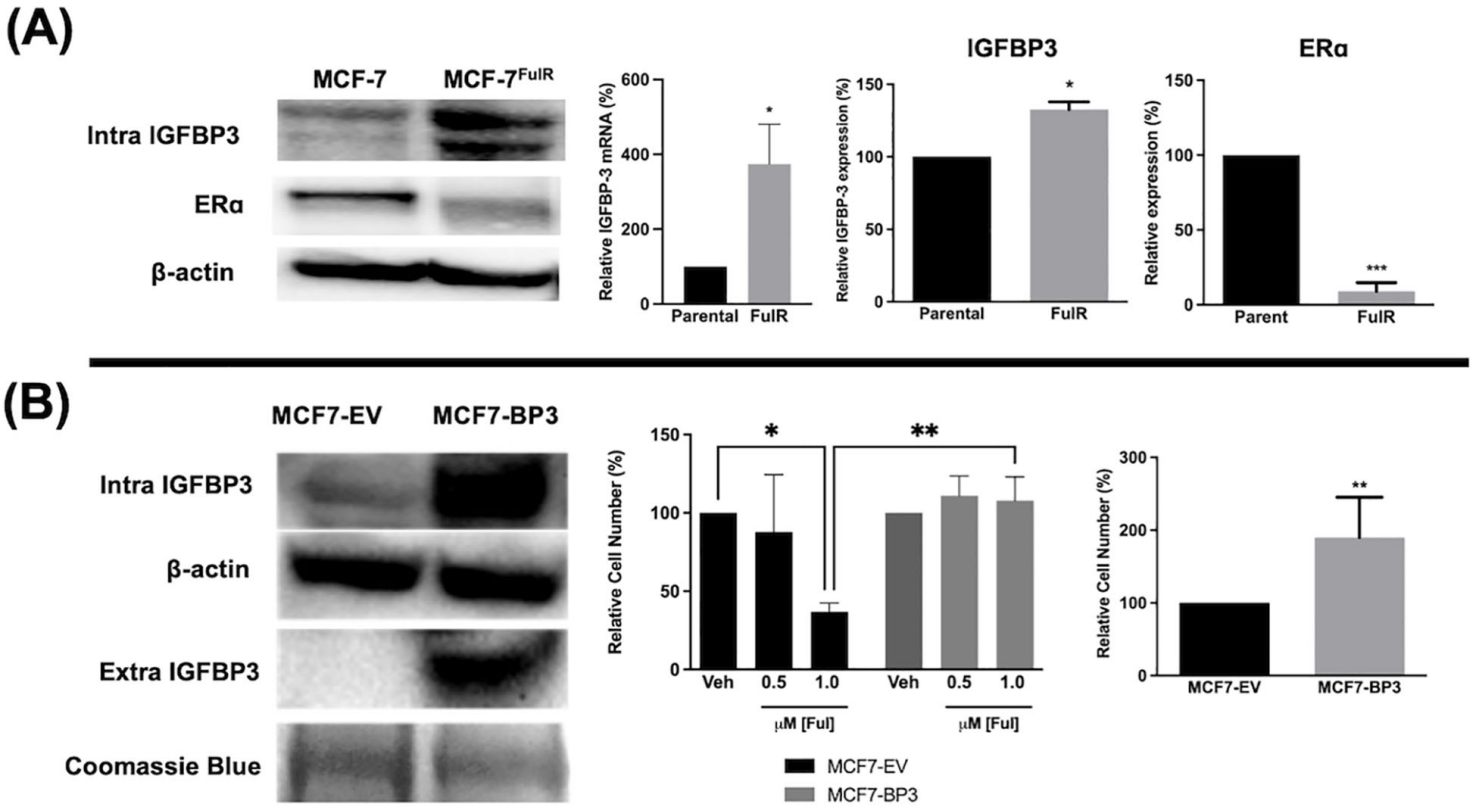
Expression of IGFBP-3 is associated with Ful resistance in MCF-7 cells. **(A)** Immunoblot analysis of IGFBP-3 and ERα in MCF-7 parental and MCF-7FulR (left), qPCR analysis of IGFBP-3 mRNA in MCF-7 parental and MCF-7FulR (right). **(B)** Immunoblot analysis of IGFBP-3 exogenous expression in MCF-7-BP3 cells (left); five-day survival of MCF-7-EV and MCF-7-BP3 cells treated with fulvestrant (middle); and five-day survival of MCF-7-EV and MCF-7-BP3 cells in maintenance media conditions (right). Bar graph results are the average of 3 independent experiments, and error bars are the standard error of the mean. *p < 0.05; **p < 0.01.

Fulvestrant is not antiproliferative in TNBC cells and this is often attributed to the lack of expression of ERα. To determine if high IGFBP-3 levels are associated with the lack of apoptotic potential of fulvestrant, Ful-resistant MCF-7 cells (MCF-7^FulR^) were generated and IGFBP-3 expression was measured. MCF-7^FulR^ cells expressed significantly higher levels of IGFBP-3 transcript and protein compared to parental cells (**Figure 2A**). To validate the resistant status of MCF-7^FulR^, the decrease in protein expression of ERα was also documented (24). Additionally, to determine if elevated IGFBP-3 expression is a determinant of Ful resistance in the MCF-7 cell line, stable subclones of MCF-7 cells expressing exogenous IGFBP-3 or empty vector (MCF-7-BP3 and MCF-7-EV, respectively) were generated and treated with Ful. After 5 days of treatment with 1 μM Ful, increased survival was observed for MCF-7-BP3 relative to MCF-7-EV cells (**Figure 2B**). In addition, increased proliferation of MCF-7-BP3 cells was observed compared to MCF-7-EV (**Figure 2B**). These data implicate IGFBP-3 in the development of Ful resistance MCF-7 cells and modulates survival and proliferation in this cell type.

### 3.3 Ful and Tam induced IGFBP-3 expression in breast cancer cells regardless of breast cancer subtype

**Figure 3:**
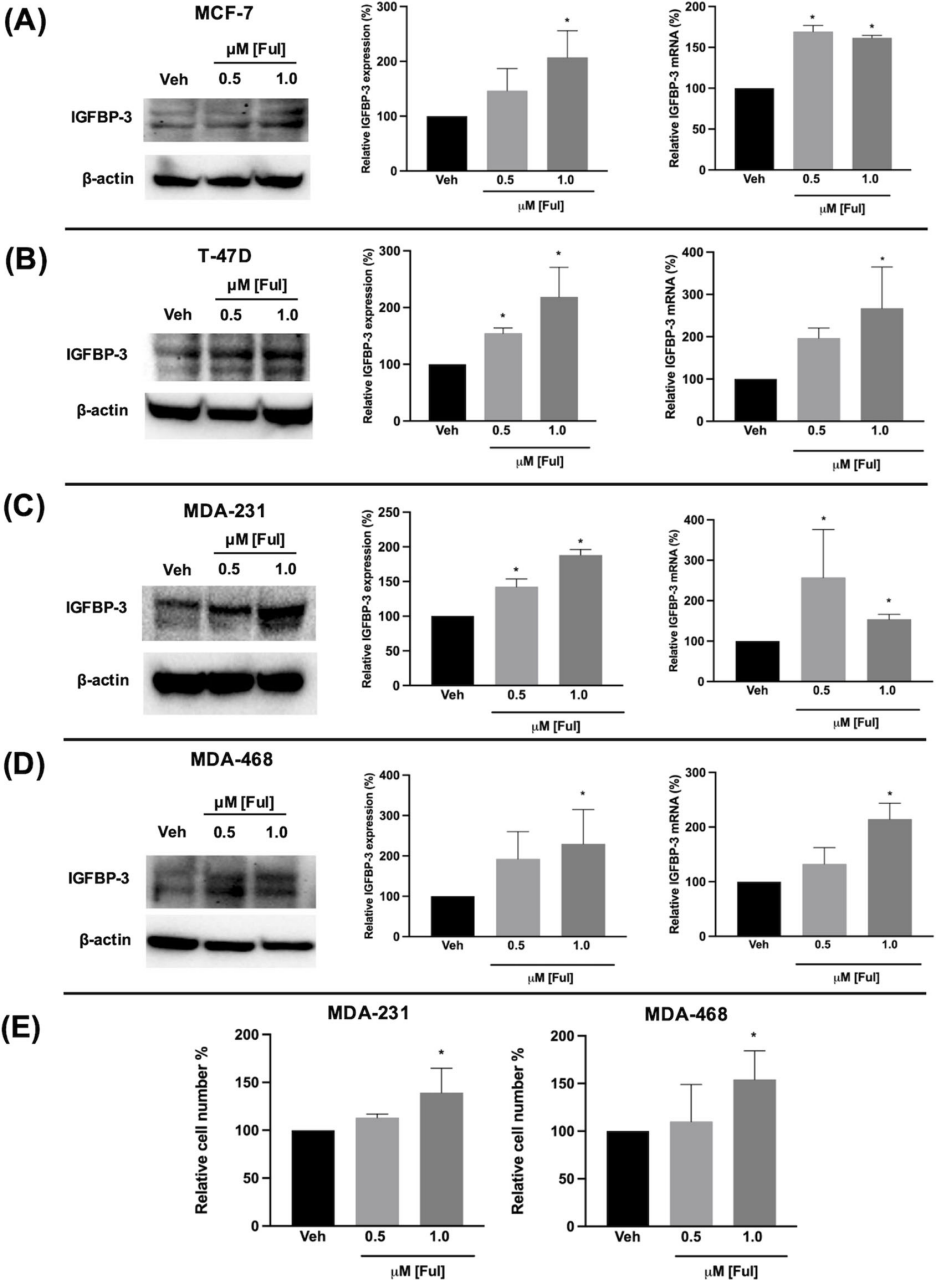
Fulvestrant induced IGFBP-3 expression in multiple breast cancer cell lines and proliferation of TNBC cells. Immunoblot analysis of IGFBP-3 and quantification by densitometry (top) and quantitative real-time PCR (middle) analysis of **(A)** MCF-7, **(B)** T-47D, **(C)** MDA-MB-231, and **(D)** MDA-MB-468 cells treated with the indicated dose of Ful. Cell proliferation was also increased in MDA-MB-231 and MDA-MB-468 cells treated with Ful after 5 days of treatment **(E)**. Results are the average of 3 independent experiments, and error bars are the standard error of the mean. *p < 0.05.

**Figure 4:**
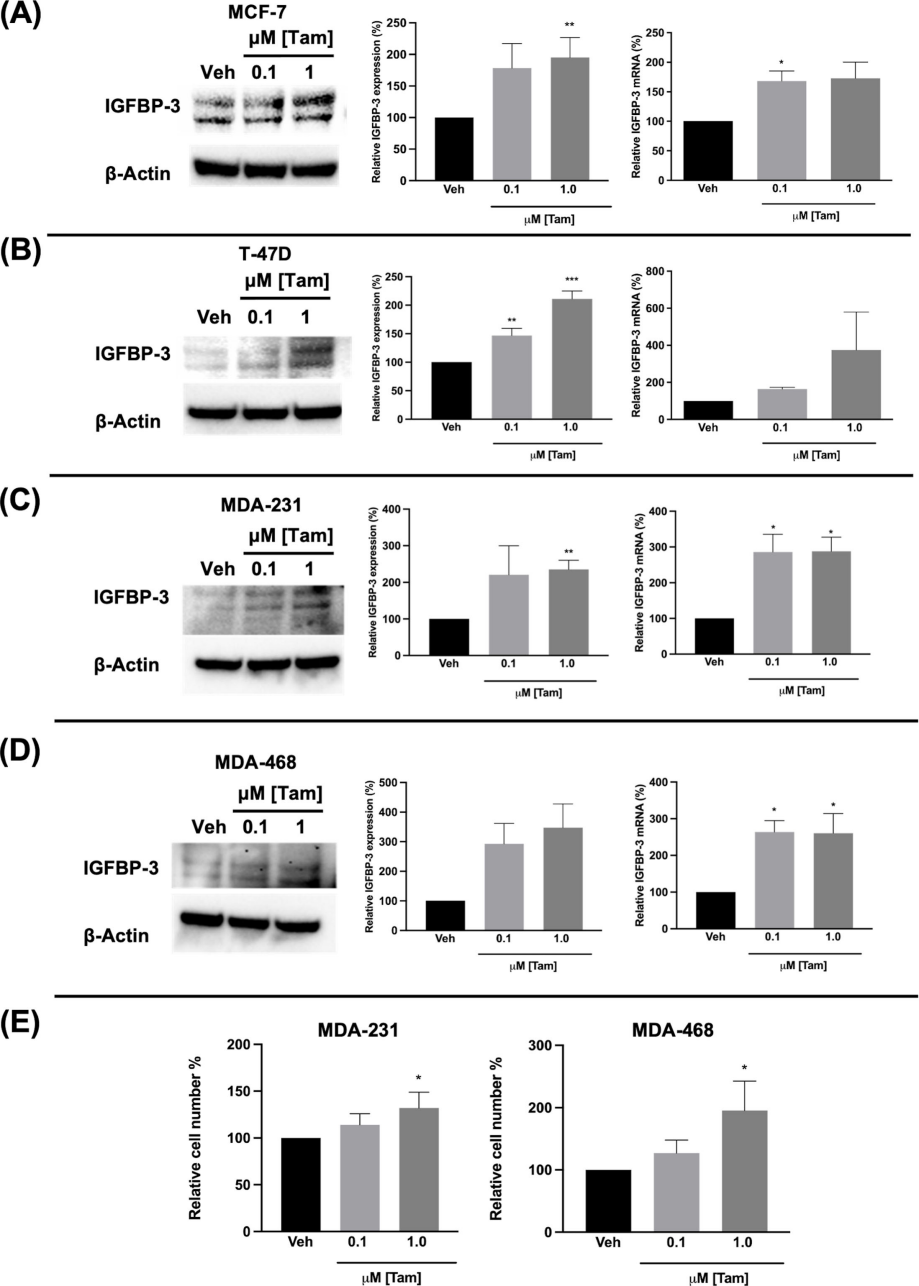
Tamoxifen also induced IGFBP-3 expression in multiple breast cancer cell lines and proliferation of TNBC cells. Immunoblot analysis of IGFBP-3 and quantification by densitometry (left) and quantitative real-time PCR (right) analysis of **(A)** MCF-7, **(B)** T-47D, **(C)** MDA-MB-231, and **(D)** MDA-MB-468 cells treated with the indicated dose of Tam. Cell proliferation was also increased in MDA-MB-231 and MDA-MB-468 cells treated with Tam after 5 days of treatment **(E)**. Results are the average of 3 independent experiments, and error bars are the standard error of the mean. *p < 0.05; **p < 0.01.

Fulvestrant has been shown to modulate IGFBP-1 levels *in vitro* and IGFBP-3 levels in the serum of breast cancer patients (17, 19). Given the clinical data observations in **Figure 1** and the higher expression of IGFBP-3 in MCF-7^FulR^ in **Figure 2**, it was hypothesized that Ful induces IGFBP-3. In every cell line tested, Ful induced IGFBP-3 transcription and protein expression (**Figure 3**). To determine whether the induction of IGFBP-3 expression is unique to Ful, the cell lines were also treated with Tam. IGFBP-3 was also induced by Tam in all cell lines tested (**Figure 4**), suggesting that these Ful and Tam may have a common mechanism of IGFBP-3 induction in breast cancer cells. Additionally, Ful and Tam induced the proliferation of MDA-MB-231 and MDA-MB-468. These data are consistent with published results suggesting that IGFBP-3 is proliferative in TNBC (13, 14).

### 3.4 GPER1 specific agonist G-1 induced IGFBP-3 accumulation and cell proliferation in TNBC cells via activation of YAP/TAZ

**Figure 5:**
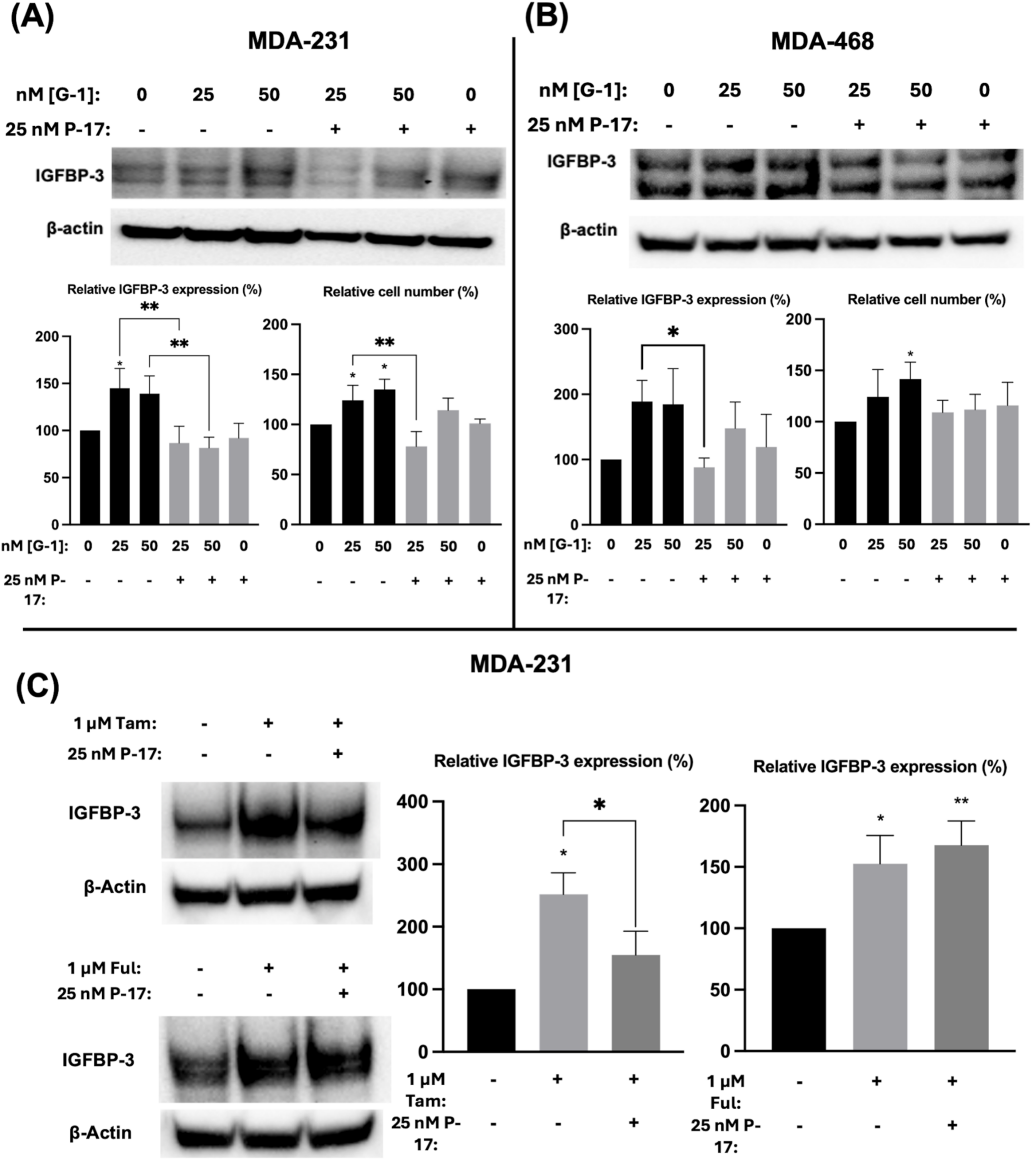
Induction of IGFBP-3 expression in TNBC occurs by a GPER1/YAP/TAZ signaling pathway. Immunoblot analysis of IGFBP-3 and quantification by densitometry, and cell number changes after treatment with the indicated dose of G1 and/or P-17 in **(A)** MDA-MB-231 cells or **(B)** MDA-MB-468 cells. **(C)** MDA-MB-231 cells were treated with 1 μM Tam or Ful and intracellular IGFBP-3 expression was detected via immunoblot; densitometry values are provided to the right of representative immunoblots. IGFBP-3 protein analysis was completed after 24-hour treatment and cell number measurement after 5-day treatment. Bar graph results are the average of 3 independent experiments, and error bars are the standard error of the mean. *p < 0.05; **p < 0.01.

Ful has previously been shown to activate the G protein-coupled estrogen receptor 1 (GPER1) in breast cancer cells (25). To determine if the observed modulation of IGFBP-3 expression was mediated by GPER1, TNBC cells were treated with G-1, the specific GPER1 agonist (18), and IGFBP-3 expression was measured. Nanomolar doses of G-1 increased the IGFBP-3 protein expression (**Figure 5**). Consistent with the IGFBP-3 protein level, G-1 also increased TNBC cell numbers. These results for G-1 treatment of TNBC cells were consistent with previously published observations (26). G-1 activation of GPER1 has been shown to activate the transcription cofactor yes-associated protein 1 (YAP) and transcriptional coactivator TAZ (27). YAP/TAZ also induces IGFBP-3 expression in mammalian cells through interaction with transcription factors TEAD 1-4 (28). To determine if G-1-induced IGFBP-3 expression depends on YAP/TAZ activation in TNBC, cells were co-treated with the YAP/TAZ inhibitor Peptide 17 (P-17) and G-1. 25 nM P-17 was sufficient to block the induction of IGFBP-3 expression in MDA-MB-231 cells, but 25 nM P-17 treatment alone did not alter IGFBP-3 expression (**Figures 5A, B**). Additionally, cell numbers were determined after G-1 treatment or G-1 and P-17 co-treatment in TNBC cells. These data showed that P-17 co-treatment blocked the stimulatory effect of G-1 on MDA-MB-231 cell proliferation. The lack of significant changes in cell number during G-1 and/or P-17 treatment observed in MDA-MB-468 cells could be explained by the already elevated levels of IGFBP-3 expression previously described in **Figure 1**. Finally, to link the GPER1/YAP/TAZ/IGFBP-3 pathway to Ful and Tam treatment, MDA-MB-231 cells were co-treated with Tam or Ful and P-17. IGFBP-3 expression in response to Tam/P-17 co-treatment decreased relative to Tam treatment in MDA-MB-231 cells (**Figure 5C**), while P-17 was unable to block the induction of IGFBP-3 by Ful (**Figure 5C**). The inconsistency of P-17-mediated inhibition of IGFBP-3 in the co-treatments suggests that Ful does not regulate IGFBP-3 expression through YAP/TAZ; however, GPER1 may regulate IGFBP-3 expression through activation of alternative downstream pathways such as CREB or ELK1 (29). Taken together, these data represent a mechanistic understanding of the regulation of IGFBP-3 in breast cancer cells by Ful and Tam.

## 4 Discussion

High expression of IGFBP-3 was observed in TNBC cells relative to antiestrogen-sensitive Erα positive cells. Conferring IGFBP-3 expression on MCF-7 cells exogenously protected them from high doses of Ful, and MCF-7^FulR^ expressed more IGFBP-3. Further, antiestrogens Ful and Tam induced IGFBP-3 expression in breast cancer cells regardless of subtype, and GPER1 promoted the expression of IGFBP-3 via activation of YAP/TAZ in TNBC cells. In MDA-MB-231 cells, Tam induced IGFBP-3 expression through GPER1 and YAP/TAZ, while YAP/TAZ was not implicated in Ful-induced IGFBP-3 expression. Taken together, these data suggest that IGFBP-3 is a key component in developing Ful resistance in breast cancer cells (**Graphical Abstract**).

The data herein suggest that Ful and Tam may induce IGFBP-3 by different mechanisms. While we anticipated that each ER modulator would induce IGFBP-3 via the same mechanisms, it is not surprising that Ful and Tam may have different cellular effects in terms of cell signaling. After all, Ful is a SERD and Tam is a SERM. While Ful, Tam, and G-1 all have described agonistic effects on GPER1. The induction of IGFBP-3 by Tam and G-1 in MDA-MB-231 cells proceeded through YAP/TAZ as evidenced by the inhibition of induction by P17. However, GPER1 can activate additional mediators of cell survival, such as extracellular signal-related kinase 1 and 2 (ERK1/2), ELK1, CREB, and *FOS* (30, 31) that may be involved in IGFBP-3 induction upon Ful treatment in breast cancer cells. The unresponsiveness of Ful-induced IGFBP-3 expression to YAP/TAZ inhibition points to the possibility of Ful-specific mechanisms of IGFBP-3 induction. Differences in the activation of GPER1 by Ful and Tam have been reported (32). Therefore, the possibility that Ful induces IGFBP-3 through GPER1 cannot be ruled out, and YAP/TAZ is not implicated in this method of activation. Further investigation is required to delineate the mechanistic differences between Ful and Tam with regard to IGFBP-3 induction.

The cellular explanation for the high levels of IGFBP-3 expression in TNBC merits further investigation. The extremely high levels of IGFBP-3 in MDA-MB-468 cells provide a valuable tool for future work but pose a limitation in attempting to see cellular effects by stimulating expression. The MDA-MB-468 cell line expresses higher amounts of GPER1 relative to MDA-MB-231 cells (**Supplementary Figure 1**) as well. While it may be possible that GPER1 is more constitutively active in this cell line, thus explaining the higher IGFBP-3 expression, the cells showed less robust changes of IGFBP-3 and cell proliferation in response to G-1 and P-17. Both of these findings conflict with previously established associations between IGFBP-3, GPER1, and aggressive subtypes (14, 33). One possible avenue of further inquiry would be to perform IGFBP-3 or GPER1 knockdowns by siRNA in MDA-MB-468 cells to assess the consequences of knockdown for antiestrogen sensitivity and gene expression. One additional modulator of IGFBP-3 is microRNA 34 (miR34), which was recently shown to downregulate IGFBP-3 in human lung epithelial cells (34) and may have a role in IGFBP-3 regulation in breast cancer epithelial cells. Of further note, Julovi et al. show that IGFBP-3 was predominantly localized in the nucleus of TNBC cells and nuclear localization of IGFBP-3 was a prognostic marker for aggressive subtypes (14). It remains possible that while the MDA-MB-231 cells express less IGFBP-3, the intracellular protein may be more active in the nucleus. The same applies to the status of GPER1 in MDA-MB-231 cells relative to 468 cells. This may explain the contradiction between GPER1 expression and less robust responsiveness to GPER1 in MDA-MB-468 cells, as localization of GPER1 plays an important role in dictating the activity of the protein (35). Future work would benefit from assessing nuclear localization of IGFBP-3 and GPER1 in both cell lines as well as investigating their relative expression of miR34, to elaborate on the existing mechanism suggested by the data presented here.

Given that the YAP/TAZ pathway is associated with the epithelial-mesenchymal transition (EMT) in breast and other tissues (36) and the depletion of YAP inhibits TNBC invasion and proliferation (37), one may infer that IGFBP-3 is a link between less aggressive ERα positive breast cancer and more aggressive TNBC and antiestrogen-resistant breast cancer. Future work would aim to show that IGFBP-3 up-regulates genes and signaling pathways involved in Ful resistance and mesenchymal phenotype. The transcriptomic profile of MCF-7-BP3 cells versus MCF-7-EV using RNAseq would provide a valuable starting point. It is hypothesized that IGFBP-3 expression will induce the transcription of important biomarkers for proliferation and aggression, such as Ki-67, an important proliferation biomarker whose level is tightly associated with breast cancer aggressiveness and ER/PR negativity (38); EPHA2, a biomarker associated with basal-like/triple-negative breast cancers (39), antiestrogen resistance (40), and EMT in MCF7-10A (41, 42); and MDM4 (aka MDMX), an oncogene that promotes ERα degradation by stabilizing the E3 ubiquitin ligase MDM2 (43). RNA-seq results would be supported by phenotypic data and qPCR of notable targets, with the goal of gaining a more detailed understanding of IGFBP-3 regulation and the role that this IGF-system modulator plays in antiestrogen resistance and TNBC etiology.

## Data Availability

The original contributions presented in the study are included in the article/**Supplementary Material**. Further inquiries can be directed to the corresponding author.
