# Supplementary figures and images for: Insulin-like growth factor-binding protein-3 is induced by tamoxifen and fulvestrant and modulates fulvestrant response in breast cancer cells
Source: Front Oncol. 2024 Nov 15;14:1452981. doi: 10.3389/fonc.2024.1452981 (PMC11604585; doi:10.3389/fonc.2024.1452981)

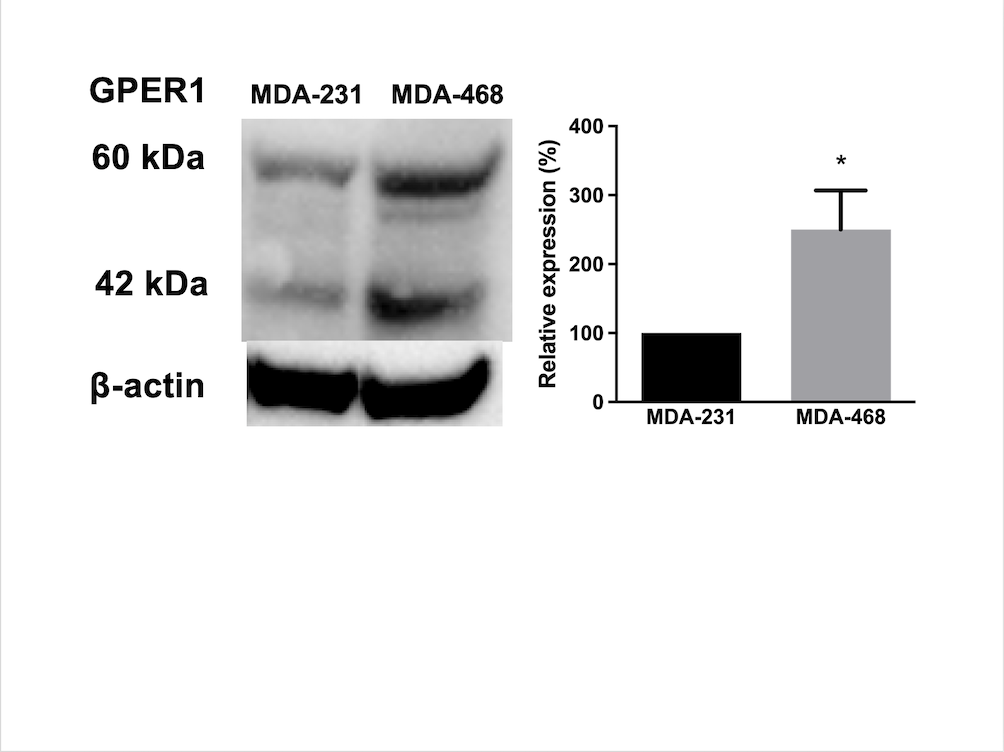

Supplement: Supplementary file 1 [file Image1.tiff]
